# Natural language processing methods are sensitive to sub-clinical linguistic differences in schizophrenia spectrum disorders

**DOI:** 10.1038/s41537-021-00154-3

**Published:** 2021-05-14

**Authors:** Sunny X. Tang, Reno Kriz, Sunghye Cho, Suh Jung Park, Jenna Harowitz, Raquel E. Gur, Mahendra T. Bhati, Daniel H. Wolf, João Sedoc, Mark Y. Liberman

**Affiliations:** 1 Zucker Hillside Hospital, Department of Psychiatry, 75-59 263rd St., Glen Oaks, NY USA; 2 University of Pennsylvania, Department of Psychiatry, 3400 Spruce St, Gates Building, Philadelphia, PA USA; 3 Linguistics Data Consortium, 3600 Market St, Suite 810, Philadelphia, PA USA; 4 University of Pennsylvania, Department of Computer Science, 3330 Walnut St, Levine Hall, Philadelphia, PA USA; 5 Stanford University, Department of Psychiatry and Neurosurgery, 401 Quarry Road, Stanford, CA USA; 6 New York University, Department of Technology, Operations, and Statistics, 44 West Fourth Street, Kaufman Management Center, New York, NY USA; 7 University of Pennsylvania, Department of Linguistics, 3401-C Walnut St, Suite 300, C Wing, Philadelphia, PA USA

**Keywords:** Psychosis, Biomarkers

## Abstract

Computerized natural language processing (NLP) allows for objective and sensitive detection of speech disturbance, a hallmark of schizophrenia spectrum disorders (SSD). We explored several methods for characterizing speech changes in SSD (*n* = 20) compared to healthy control (HC) participants (*n* = 11) and approached linguistic phenotyping on three levels: individual words, parts-of-speech (POS), and sentence-level coherence. NLP features were compared with a clinical gold standard, the Scale for the Assessment of Thought, Language and Communication (TLC). We utilized Bidirectional Encoder Representations from Transformers (BERT), a state-of-the-art embedding algorithm incorporating bidirectional context. Through the POS approach, we found that SSD used more pronouns but fewer adverbs, adjectives, and determiners (e.g., “the,” “a,”). Analysis of individual word usage was notable for more frequent use of first-person singular pronouns among individuals with SSD and first-person plural pronouns among HC. There was a striking increase in incomplete words among SSD. Sentence-level analysis using BERT reflected increased tangentiality among SSD with greater sentence embedding distances. The SSD sample had low speech disturbance on average and there was no difference in group means for TLC scores. However, NLP measures of language disturbance appear to be sensitive to these subclinical differences and showed greater ability to discriminate between HC and SSD than a model based on clinical ratings alone. These intriguing exploratory results from a small sample prompt further inquiry into NLP methods for characterizing language disturbance in SSD and suggest that NLP measures may yield clinically relevant and informative biomarkers.

## Introduction

Language disturbance has long been recognized as a hallmark of psychosis, ranging from marked disorganization in threshold schizophrenia spectrum disorders (SSD) to less pronounced phenotypes among subthreshold psychosis-spectrum conditions like schizotypy, clinical high risk, and genetic risk for psychosis^1–5^. Speech is considered the observable surface phenomena that reveal, in part, the shrouded thoughts of the inner “mind.” Thus, thought disorder in psychosis has often been equated with speech disturbance^6^. Novel digital phenotyping and computerized natural language processing (NLP) methods offer the opportunity to capture speech in ecologically valid settings and automatically quantify objective parameters that reflect underlying thought disturbance^7^. This would enable substantial advancements in assessment and monitoring of treatment response and could be used in research to probe more deeply into the psychosis disease process. However, a great deal remains to be understood about how to best leverage both established and state-of-the-art NLP tools to tease out clinically meaningful linguistic parameters that will have diagnostic and prognostic value.

A variety of NLP techniques have been used to characterize different language phenotypes in psychosis with varying degrees of success, mostly focusing on SSD^8^. Here, “schizophrenia spectrum disorders” (SSD) refers to threshold-level primary psychotic disorders^9,10^. Elvevåg et al.^11^ first applied Latent Semantic Analysis to quantify decreased coherence in SSD speech and was able to predict human ratings of organizational structure, tangentiality, and content, as well as discriminating between SSD and control participants with 80–82% accuracy. Several subsequent studies have also modeled decreased linguistic cohesion and found significant group differences between SSD and control participants^12,13^. Decreased semantic coherence may predict conversion to psychotic disorders among young people at clinical risk for psychosis with 70–80% accuracy^5,14^. Other successful approaches have included graph analysis^15,16^, quantifying semantic density^17^, and automated metaphor detection^18^. In one study, NLP analysis of semantic cohesion accounted for 10% of variance in neurocognition beyond clinician ratings^19^. Another study used automated speech recognition and scoring on a smartphone verbal memory test to accurately predict human ratings^20^. Multiple levels of analysis including generic features like number of words per sentence, individual word identity, dictionary features reflecting word categories, and language model features like n-grams were incorporated in another study to discriminate between SSD and control speech with 74% accuracy^21^.

However, other studies have demonstrated negative or conflicting results, suggesting that a great deal remains to be understood about applying NLP to evaluate speech disturbance in psychosis. Despite affective flattening being a common negative symptom in SSD, use of emotion words were nevertheless similar between SSD and control participants^22^. Cohesion was not significantly reduced in another study comparing first-episode psychosis and healthy control (HC) participants^23^. Multiple levels of acoustic and linguistic analysis were shown to have a poor correlation with clinician ratings for negative symptoms^7^. In general, despite seemingly straightforward analogies between acoustic features like pitch variability and pause duration with negative symptoms like affective flattening and alogia, NLP measures have shown poor ability to track negative symptoms, including affective flattening and alogia^24^. Existing studies in psychiatry also use older NLP methods that have been supplanted by more advanced methods in the computational field. In particular, previous work on categorizing psychosis spectrum language samples^12,14^ have leveraged non-contextual word embeddings such as GloVe and Word2Vec^25^. For example, these older NLP methods are unable to incorporate contextual cues that would distinguish between the word “banks” in “I rob banks” and “I walked along the banks.”

In this study, we explored multiple NLP methods for characterizing speech changes in SSD and approached linguistic phenotyping on three levels: individual words, parts-of-speech (POS), and sentence-level measures of coherence. The NLP linguistic parameters were compared with a clinical gold standard, the Scale for the Assessment of Thought, Language and Communication (TLC)^26^, in their ability to discriminate between speech samples from SSD and HC participants. To our knowledge, we are the first to apply Bidirectional Encoder Representations from Transformers (BERT) to a psychiatric sample; BERT is a state-of-the-art embedding architecture that is trained to incorporate bidirectional context and predict whether two sentences are adjacent^27^. This advance is significant because it allows us to look at larger units of discourse, and provides more accurate representation of the concepts being conveyed—treating discourse as an ordered series of information exchanges with a direction rather than an unordered collection of phrases. Moreover, BERT has been shown to be superior to other methods on several NLP tasks^27^. The samples were not enriched for overt thought or language disorder, in order for the speech and language phenotype to be more representative of the range present in SSD as a whole. Because this was an exploratory study and because no consistent set of key NLP predictors have emerged from prior work, we did not approach the analyses with a priori expectations of specific features that would be associated with SSD. Instead, we broadly hypothesized that speech from individuals with SSD would show abnormalities on each level of analysis: individual words, POS, and sentences. In addition, we expected that NLP would be better able to discriminate between SSD and HC language than the gold standard clinical rating scale for language disturbance. Of note, we encountered unexpected findings regarding incomplete words in SSD and potential methodological pitfalls, which are also detailed below.

## Results

### Clinical ratings of language disorder

**Fig. 1:**
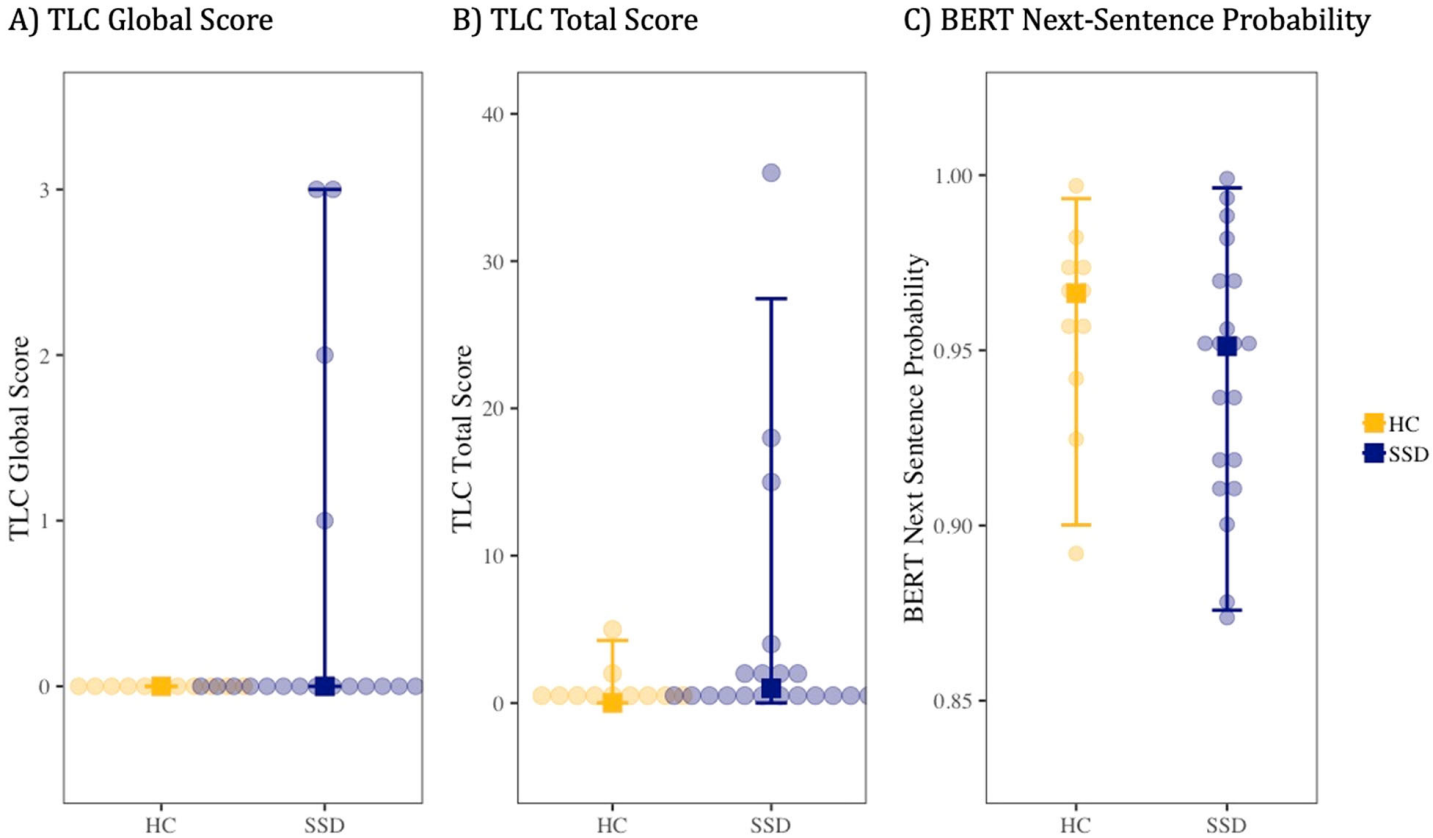
Group effects on clinical language ratings and BERT next-sentence probability. Individual scores with group median and interquartile range are shown. There were no significant group differences for (**A**) TLC Global Score (Cohen’s *d* = 0.55, *p* = 0.13), (**B**) TLC Total Score or Sum (Cohen’s *d* = 0.48, *p* = 0.10), and (**C**) BERT Next-Sentence Probability (Cohen’s *d* = −0.44, *p* = 0.25). The three SSD participants with TLC global scores ≥2 were identified as outliers.

Individual TLC items are detailed in Supplemental Table 2. Clinically significant language disorder was present in four participants with SSD and none of the HC participants. Three of the SSD participants with TLC global score ≥2 were identified as outliers in this sample. However, there were no significant group differences in the TLC global, sum, or any individual item score (Table 1, Fig. 1). The largest effect sizes were in poverty of content of speech (Cohen’s *d* = 0.70) and illogicality (Cohen’s *d* = 0.51).

**Table 1:** Sample Characteristics.

	HC	SSD	*p* value	Cohen’s *d*
**Sample**				
*n*	11	20		
Cohort			0.10	
Cohort 1	5	15		
Cohort 2	6	5		
Age (mean years ± SD)	35.6 ± 5.8	36.5 ± 7.2	0.75	0.12
Sex (*n*, %)				
Female	7 (64%)	9 (45%)	0.32	
Male	4 (36%)	11 (55%)		
Race (*n*, %)			0.12	
African American	3 (30%)	13 (65%)		
Asian	0 (0%)	1 (5%)		
Caucasian	7 (70%)	6 (30%)		
Education level	15.8 ± 2.2	13.4 ± 2.5	0.01	−1.00
**Recording** **Characteristics**				
Recording duration (min)	11.6 ± 2.2	12.7 ± 4.5	0.48	0.29
Mean sentence length (words)	17.5 ± 3.1	14.4 ± 4.3	0.04	0.81
Word count	1748.8 ± 448.0	1782.3 ± 908.2	0.92	0.04
**Language** **Measures**				
TLC Global Score	0.0 ± 0.0	0.5 ± 1.0	0.13	0.56
TLC Total Score	0.9 ± 1.7	4.4 ± 9.2	0.10	0.46
Next-sentence predictability	0.96 ± 0.03	0.94 + 0.04	0.25	-0.44

TLC global score is an overall impression of speech and language disturbance based on standard anchors. TLC total score is summed using the published formula, Total = 2*(Sum of items 1–11) + (Sum of items 12–18). Next-Sentence Predictability derived from BERT, with 0 indicating low predictability and 1 indicating high predictability. Additional details about the SSD participants are provided in Supplemental Table 1.

**Bolded** – categories; *HC* healthy control participants, *SSD* participants with schizophrenia spectrum disorder, *TLC* Scale for the Assessment of Thought Learning and Communication (Andreasen^26^).

### NLP measures for individual words and POS

SSD and HC employed distinct word usage patterns, with top SSD- and HC-associated words listed in Supplemental Table 3. Of note, top SSD-associated words included first-person singular (“I,” etc.) and second-person pronouns (“you,” etc.), the filler word “uh,” and incomplete words. Top HC-associated words included first-person plural pronouns (“we,” etc.), the filler word “um,” and laughter. Supplemental Fig. 1 further details the propensity for participants with SSD to use the first-person singular over plural pronouns, and the filler word “uh” over “um.” The use of incomplete words, on its own, was able to discriminate between SSD and HC groups with AUC = 0.88, accuracy = 90% (Supplemental Fig. 2A). With leave-one-out cross validation, word usage alone discriminated between SSD and HC with AUC = 0.80, accuracy = 76% (Supplemental Fig. 2B).

Mean POS counts per 100 words and group comparisons while covarying for demographic variables, education level, and cohort are reported in Table 2. Participants with SSD produced fewer adverbs (*p* = 0.001), adjectives (*p* = 0.03), and determiners (*p* = 0.03) but produced more pronouns (*p* = 0.03). The group differences in the other POS categories were not significant, including for interjections as a whole (despite differences for the specific filler words, “um” and “uh”). Excluding the three SSD outliers, the group differences in adverbs (*p* < 0.001), adjectives (*p* = 0.04), and determiners (*p* = 0.03) remained but the difference in pronouns became trend-level (*p* = 0.09).

**Table 2:** Parts-of-speech frequencies in SSD and HC.

	HC (*N* = 11)	SSD (*N* = 20)	*p*-value	Cohen’s *d*
Adverb	10.65 (0.95)	8.11 (1.76)	0.001	1.66
Determiner	7.50 (0.96)	6.53 (1.25)	0.03	0.83
Adjective	7.10 (1.57)	6.19 (0.78)	0.03	0.82
Pronoun	11.77 (1.47)	13.41 (2.67)	0.03	−0.71
Preposition	8.84 (1.37)	7.97 (1.41)	0.08	0.62
Particle	2.65 (0.52)	2.35 (0.50)	0.08	0.59
Conjunction	5.33 (1.35)	4.61 (1.40)	0.29	0.53
Noun	13.16 (0.93)	13.67 (2.16)	0.57	−0.28
Interjection	6.07 (1.66)	6.35 (2.45)	0.75	−0.12
Verb	19.34 (1.74)	19.55 (2.60)	0.82	−0.09

*HC* healthy control participants, *SSD* participants with schizophrenia spectrum disorder, *Adverb* word that modifies an adjective or verb, *Determiner* determines the kind of reference for a noun, e.g. the, this, a, *Adjective* word that modifies a noun, *Pronoun* word that refers to the self or another noun mentioned elsewhere, e.g., I, she, them, *Preposition* word expressing relation to another clause, e.g. on, after, for, *Particle* function word providing meaning to associated words, e.g., **to** run, ate **up**, talk **over**, *Conjunction* connector word, e.g., and, but, because, *Noun* a person, place, thing, state, or quality, *Interjection* utterance expressing emotion, e.g., ouch, ugh, hey, *Verb* word expressing action, state, or relation.

*P*-values shown for ANCOVA tests, co-varying for education level, study cohort, and demographic variables (age, sex, and race).

### Sentence-level NLP results

**Fig. 2:**
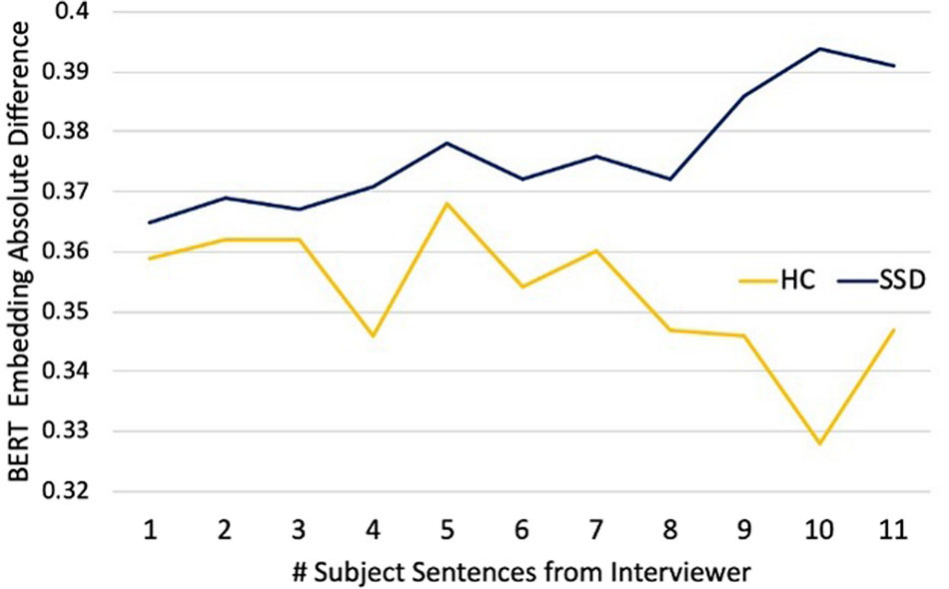
Sentence embedding distance by interviewer-participant exchanges. The average BERT sentence embedding difference between the original interviewer prompt and the participant’s response sentence, varying by distance from the original prompt. Responses from individuals with SSD began significantly farther from interviewer prompts relative to HC and traveled increasingly father away where those of HC did not. Fitting linear regressions to the trajectories, we find: SSD intercept = 0.260, 95% CI [0.257, 0.263]; SSD slope = 6.6e−4, 95% CI [2.6e−4, 1.1e−3]; HC intercept = 0.247, 95% CI [0.242–0.252]; HC slope = 1.5e−5, 95% CI [−6.2e−4, 6.5e−4]. The analysis was repeated excluding the 3 SSD outliers with high TLC scores and the results were consistent.

In a linear regression model covarying for demographic variables, education level, and cohort, group failed to predict mean BERT next-sentence probability scores (Table 1; Beta coefficient = 0.01, *p* = 0.28). Weighting by normalized sentence length and exclusion of very brief sentences (less than five words) did not alter the null results. Figure 2 shows the average BERT embedding difference between the original interviewer prompt and the participant’s response sentence, varying by distance from the original prompt. Modeling the trajectories for embedding distance, we found the intercept for SSD (0.260, 95% CI [0.257, 0.263]) was significantly higher than of HC (0.247, 95% CI [0.242–0.252]), suggesting that responses were more unrelated to interviewer prompts. Furthermore, the slope of the SSD embedding trajectory was significantly above zero (6.6e−4, 95% CI [2.6e−4, 1.1e−3]) but the slope for HC was not significantly different from zero (1.5e−5, 95% CI [−6.2e−4, 6.5e−4]), suggesting that SSD sentences diverged significantly from interviewer prompts while HC sentences did not. Omitting the 3 outliers from the analysis did not alter this pattern.

### Comparing clinical and NLP language measures

**Fig. 3:**
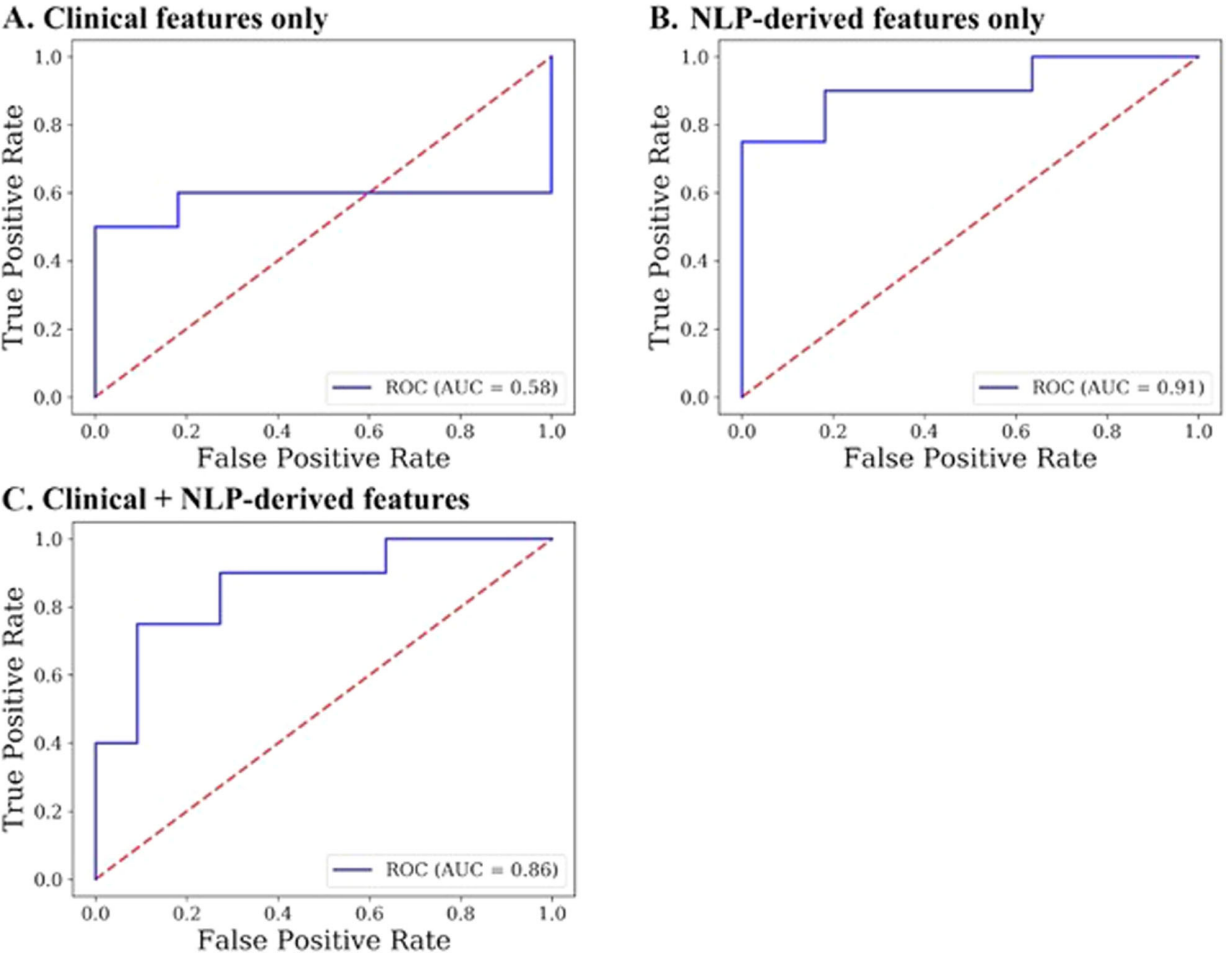
Discrimination between SSD and HC group status. Naive Bayes models with leave-one-out cross-validation were constructed for (**A**) Clinical features alone, from the Scale for the Assessment of Thought Language and Communication (TLC), (**B**) Natural language processing-derived linguistic features alone, and (**C**) A combination of TLC and NLP-derived features. The NLP-only model performed better than the clinical-only model (accuracy 87% compared to 68%) and was similar to the model incorporating both NLP and clinical linguistic features (accuracy 81%).

Figure 3 shows receiver operative characteristics for naive Bayes models discriminating between SSD and HC groups based on language measures. Using leave-one-out cross-validation, area under the curve (AUC) for clinical measures alone (0.58, Accuracy = 68%) was substantially lower than for the model based on NLP measures alone (AUC = 0.91, Accuracy = 87%). Adding clinical measures to the NLP model did not improve discriminating ability (AUC = 0.86, Accuracy = 81%). The inclusion of education level as an additional predictor did not substantially change the accuracy of these models—NLP alone (Accuracy = 87%) and NLP + TLC measures (Accuracy = 77%) continued to outperform the model based on TLC measures alone (Accuracy = 68%). We also confirmed these results using fivefold cross validation and found a similar pattern, and higher accuracy. There was insufficient variability in clinical ratings to explore the correlation between TLC and NLP measures.

## Discussion

In this exploratory study, we applied NLP to multiple levels of linguistic analysis comparing speech among SSD and HC participants. Individuals with SSD used significantly more pronouns and made more speech errors/partial words but used fewer adverbs and determiners. Analysis of individual word usage was notable for more frequent use of first-person singular pronouns among individuals with SSD and more frequent use of first-person plural pronouns among HC. The filler “uh” was highly associated with SSD, while “um” was associated with HC. There was also a striking increase in incomplete words among the SSD group. Sentence-level analysis using BERT reflected potentially increased tangentiality among SSD; sentence embedding distance increased for participants with SSD more than for HC. Participants with SSD generally had low language disorder and group means were not distinguishable from HC based on blinded clinical TLC ratings. It is unclear whether the specific linguistic group differences reported here represent clinically relevant, generalizable features of language disturbance in SSD. However, on the whole, NLP measures of language disturbance appear to be sensitive to these subclinical differences as NLP-based models had a greater ability to discriminate between HC and SSD than a model based on clinical ratings—whether or not education level was included as an additional predictor.

Differences in pronoun usage have been noted by several other studies. Buck et al.^28^ also found increased pronoun use overall and increased first-person singular pronoun use among individuals with SSD when compared to HC. While the differences were not significant, they found similar trends with increased use of second-person pronouns among SSD and increased use of first-person plural pronouns among HC^28^. Increased use of first-person pronouns was also found among written text samples from individuals with SSD compared to HC^29^. Another study found significantly increased use of second-person pronouns among individuals with genetic risk for schizophrenia who later developed schizophrenia, compared to those who did not^30^. These differences in pronoun use appear to be context specific. Analysis of Twitter language found increased use of first-person singular and plural pronouns among posts from individuals who self-identified as having schizophrenia^31^. When compared to individuals with depression, those with SSD used fewer first-person singular pronouns^32^. Further delineation of differences in pronoun use is necessary as this is among the more highly replicated objective language disturbances in SSD and may reflect informative underlying differences in self-concept and/or social and metacognition^28,32^.

“Um” and “uh” are frequently referred to as filled pauses, but may also be considered interjections signaling a preparedness problem on the part of the speaker and an impending delay^33,34^. “Um” takes a longer time to pronounce and usually signals a longer delay than “uh”^33^. The overall occurrence of filled pauses in speech from individuals with schizophrenia was previously found to be similar to that of neurotypical controls, despite greater occurrence of unfilled pauses or blocking prior to initiation of a new clause^34,35^. Our finding of greater “uh” occurrence among SSD and greater “um” occurrence among HC has not been previously reported, to our knowledge. In fact, these filler words are often excluded from analysis^12^. However, interestingly, a similar difference in filler word usage has been described among children with autism spectrum disorder with individuals with ASD using a higher proportion of “uh” compared to “um”^36^. While any connection between these findings is purely speculative at this point, it does raise the fascinating possibility that shared changes in brain circuitry may lead to a preference for “uh” over “um” among individuals with SSD and ASD. It remains to be determined whether the greater prevalence of “uh” spoken by SSD corresponds to a greater prevalence of short pauses, compared to long pauses, or whether this difference in “um”/”uh” usage is a symptom of impaired or inaccurate signaling by speakers with SSD, dissociated from pause duration.

Similarly, we are the first to report an increase in incomplete spoken words among individuals with schizophrenia. While decreased fluency has been noted in general, to our knowledge, the phenomenon of stopping mid-word has not been discussed in the literature in relation to SSD despite our finding that this one feature discriminates between SSD and HC with AUC 0.88. It is likely that this finding has not been replicated because these partial words were often considered meaningless and therefore not transcribed or discarded from the analysis. In live conversations, there is also a cognitive propensity not to notice these disfluencies^37^. If this finding is replicated, the use of incomplete words may provide a powerful objective marker of language disturbance and language circuity abnormality among individuals with SSD.

In this study, NLP measures alone were better able to discriminate between SSD and HC than clinical TLC ratings. The prediction model achieved 87% accuracy with leave-one-out cross validation. This accuracy is similar to results from other computational linguistics analyses, which have been reported at 69%^14,16^, 69–75%^38^, 83%^39^, 84%^18^, and 87–93%^12^. Advances in this field may contribute substantially to our understanding of the neurobiological underpinnings of schizophrenia. NLP measures have been tied to specific cognitive deficits in schizophrenia-like attention and social cognitive impairments^28,40,41^. Outside of psychiatry, NLP measures from healthy adults predicted stress response-related gene expression with better accuracy than self-reports^42^. To better understand the pathological mechanisms of psychiatric disorders and to develop targeted treatments, quantitative objective biomarkers of changes in brain circuitry are sorely needed.

There are several limitations to this study. The sample is small and heterogeneous with respect to study conditions between cohorts, with a higher n of SSD participants in Cohort 1 than Cohort 2, accounting for 48% of the total sample. However, results did not differ when we covaried for cohort. We were powered for differences with large group effects and are likely to miss small and medium effects. Due to the exploratory nature of this investigation, we did not correct for multiple comparisons, and therefore some findings may be the result of “overfitting” and may not be generalizable. The low number of individuals with clinically evident thought disorder limited our ability to assess correlations between NLP and TLC measures, but also demonstrates the ability of NLP measures to detect subclinical language disturbances in SSD. As a field, careful attention should be paid to technical quirks which can bias results, like our experience with automated sentence parsing resulting in falsely decreased coherence in the SSD group (as measured by BERT next-sentence probability). Other groups have had similar experiences^12^. Some of these errors result from the fact that most NLP tools have been trained on written texts, not speech. There is a need for a large shared corpus of clinical speech samples to advance NLP methods in psychiatry research. Since BERT is specifically trained on sentence-to-sentence prediction, this was the most straightforward and natural way of applying BERT to the coherence task and within-sentence coherence was not examined in this study. Furthermore, prior studies suggest that medication effects may drive some language abnormalities in SSD, particularly impaired phonation, as well as decreased quantity and diversity of words spoken^43,44^. Future efforts should assess how NLP measures correlate with specific dimensions of psychosis symptoms and medications used, for which consistent data were not available in these cohorts.

To our knowledge, this is the first study to apply BERT or any state-of-the-art embedding method that incorporates bidirectional context. Given the limited sample size in this exploratory study, findings should not be considered definitive or generalizable. However, our results nevertheless prompt further inquiry into NLP methods for characterizing language disturbance in SSD and suggest that NLP measures as a whole may yield clinically relevant and informative biomarkers.

## Methods

### Sample

Two cohorts are included and are described in Table 1 with further details on the SSD group given in Supplemental Table 1. Both cohorts were recruited at the University of Pennsylvania and underwent written informed consent for the research procedures. Study procedures were approved by the Institutional Review Board at the University of Pennsylvania. SSD participants were stable outpatients and underwent semi-structured diagnostic interviews and consensus diagnostic case conferences which conferred a diagnosis of schizophrenia or schizoaffective disorder under DSM IV criteria^45^. Individuals with diagnosed intellectual disabilities were excluded from both cohorts. There was no selection or enrichment of the sample for participants with thought or language disorder—i.e., we did not preferentially include individuals with clinically evident symptoms of speech disorganization. HC participants underwent the same diagnostic interviews and were determined to be free of major psychiatric disorders. Both cohorts provided recorded speech samples derived from open-ended interviewing. Cohort 1 was asked to talk about themselves. Cohort 2 was asked to recount positive and neutral memories. Cohort 1 recordings were significantly shorter than those of Cohort 2 when considering total participant and interviewer speech and pause duration (10.9 ± 1.7 vs. 15.5 ± 5.4 min, *p* = 0.002), but the difference in participant word count was not significant (1661 ± 567 vs. 1970 ± 1161 words, *p* = 0.32). There were no significant differences between the cohorts in participant age, sex, education level, mean sentence length or number of sentences spoken.

Comparing the SSD and HC groups, SSD participants had significantly lower mean education level than HC. This difference was accounted for in the statistical analyses. Language samples from SSD and HC were of similar duration and total word count, but HC used longer sentences than SSD.

Recordings were transcribed verbatim by human annotators for NLP analysis, including noting non-verbal vocalizations like laughter and disfluencies like “um.” Transcriber v.1.5.2^46^ was used to transcribe audio samples from Cohort 2, while samples from Cohort 1 were transcribed using standard word processing software. To protect participant privacy, all personal or identifying references to an individual or location’s name were replaced with “[name]” and all dates replaced with “[date].”

### Clinical assessments

Recordings and transcripts were reviewed by a blinded psychiatrist with significant experience with schizophrenia research and treatment (SXT). The Scale for the Assessment of Thought, Language and Communication (TLC) was used to rate participant speech on 18 items, as well as a global measure of language disorder (ranging from 0-Absent to 4-Extreme) and a summation score calculated as per the published formula detailed in Supplemental Table 2^26^. The TLC was chosen because it is freely available, highly cited, and includes a wide span of clinically evident linguistic features. The modal global severity score was 0 for both HC and SSD, reflecting generally mild or absent language disorder in the SSD group, likely because they were composed of relatively high-functioning individuals who are motivated to participate in research.

### NLP analyses for individual words and POS

Individual words were identified and utilization was compared between SSD and HC language samples. To capture larger trends, all pronouns of the same type were considered together. For example, “I” “my” and “me” were all counted as first-person singular pronouns. All incomplete words where the participant began to say a word but stopped before finishing were noted in aggregate. Odds ratios were calculated for words spoken by individuals with SSD relative to HC. The odds ratios were log-transformed and underwent weighting based on informative Dirichlet prior^47^, which takes into account the expected frequency of each word in a random text and selects for words that are more “unique” to these documents.

POS in participant speech were automatically tagged for all tokenized, individual words with spaCy, using their basic model (‘en_core_web_sm’) for English^48^. Token counts for each POS category were calculated per 100 words and compared between the SSD and HC groups.

### NLP sentence-level analyses using BERT

A major recent innovation in NLP has been the creation of large-scale pre-trained language models^27,49,50^. The most relevant pre-trained model for this work is known as Bidirectional Encoder Representations from Transformers, or BERT^27^. Unlike previous non-contextual embedding models, BERT allows for richer word representations, which takes the context surrounding a word into account. Trained on the English Wikipedia (2.5 billion words) and the Google BooksCorpus (800 million words), BERT operates with two general language understanding tasks: (1) Masked language modeling, where 15% of words from an input text are removed at random, and BERT predicts these words, and (2) Next-sentence prediction, where given two sentences, BERT predicts the likelihood of the second sentence following directly after the first.

We take advantage of the directionality incorporated into BERT next-sentence prediction to explore sentence-level coherence. For example, the BERT next-sentence probability for the below sentence pair is 1.0; this means the model is extremely confident that sentence 2 directly follows sentence 1. On the other hand, if the order of the sentences is reversed, the next-sentence probability is instead 0.002.

“Um, what do you think about current political issues like the energy crisis?”

“They’re destroying too many cattle and oil just to make soap.”

### Excerpted from an example of language disturbance in the TLC Scale^26^

In this work, we employed two BERT-based methods to compare sentence-level differences between SSD and HC language samples, one based on next-sentence predictability, and the other based on BERT embeddings. In the next-sentence predictability approach, we performed sentence splitting on the interviews by using NLTK^51^, which split sentences at intuitive punctuation points identified by transcribers. Sentence pairs were extracted from each interview, where the second sentence of each pair was uttered by the interview subject. From there, we directly leveraged BERT to predict the next sentence probability of each pair. Weighting the next-sentence probabilities by sentence length was attempted but did not alter the results. Note that initially, we used SpaCy to automatically split interview turns into sentences^48^. However, upon closer inspection, we discovered that this was incorrectly segmenting sentences at incomplete, filler, and repeated words. These segments (e.g., “I sto- // stopped at the store”) were more frequent in SSD and were assigned low BERT next-sentence predictability scores and biased initial results toward a significant difference in mean next-sentence predictability. The results presented here instead used sentences segmented intuitively by transcribers, which considered filler words and repeated phrases as a part of the same sentence.

In the embedding approach, we broke down the interviews into full dialogue turns (exchanges beginning with an interviewer prompt and followed by any number of participant sentences until the interviewer speaks again for the next turn). We generated a single embedding *e*_*i*_ for the entire Interviewer dialogue turn *t*_*i*_; we did this by generating BERT embeddings for each word in *t*, and computed the mean embedding across all words. From there, we generated a sentence-level BERT embedding *e*_*sj*_ for each sentence *s*_*j*_ in the subsequent subject turn and calculated the mean difference between *e*_*i*_ and *e*_*sj*_. The intuition behind this is that if tangentiality or derailment is present, participant responses are likely to move further away from the initial interview prompt compared to coherent exchanges. Simple linear regression models were fit on sentence-wise embedding distance to compare the slopes and intercepts of the embedding trajectories for the two groups. As BERT is specifically trained on sentence-to-sentence prediction, our analyses were done on the sentence-level and within-sentence incoherence was not analyzed.

### Statistical analysis

Normality was assessed with the Shapiro-Wilkes test and significant departure from normality was found for TLC global score (*p* < 0.001) and TLC sum (*p* < 0.001). Three outliers in the SSD group with high TLC scores were identified with the standard boxplot method. Group differences for these measures were compared with the Wilcoxon Rank Sum test with two-sided *alpha* = 0.05. Other measures were also tested for normality and we found they met the requirements for parametric tests. We built ANCOVA models for group comparisons of POS, covarying for education level, cohort, and demographic variables (age, sex, and race). All effect sizes were measured with Cohen’s *d*. BERT next-sentence probability was calculated for each participant by taking the mean of the BERT probability scores for every sentence uttered by the participant, directly from the BERT output. To evaluate group effects, linear models were constructed with mean BERT next-sentence probability as the outcome variable and group as predictor while covarying for cohort, education level, and demographic variables (age, sex, education level). Due to the exploratory nature of these analyses, we did not correct for multiple comparisons.

In order to test the discriminating ability of the features, we trained three different naive Bayes models from the scikit-learn package^52^, varying the number of features. The naive Bayes model was chosen because predictors were assumed to be independent, as is common in statistical modeling, and we assumed that we know something about the distributions for the outcomes independent of the predictors. One model was trained with TLC ratings only, and another model was trained with linguistic features only (log-odds ratio, BERT scores, POS counts per 100 words, and number of incomplete words). The other model was trained with the linguistics features and the TLC sum ratings. In all models, we imputed missing values with the simple imputer function, and standardized the features with the StandardScaler function for effective learning. To assess for the effect of education level on model accuracy, we additionally ran these models while also including education level as a predictor. Leave-one-out cross-validation was chosen to minimize bias and used to evaluate the general performance of the models and report AUC and the mean accuracy scores in the results.

## Supplementary information

Supplemental Materials

## Data Availability

The datasets analyzed during the current study are not publicly available due to participant privacy and security concerns, including HIPAA regulations. Qualified researchers may contact the corresponding author for access.

## Code availability

The code used in this study is publicly available at https://github.com/rekriz11/nlp_schizophrenia. Files with the extension “.Rmd” were run in R v.4.0.3, files with extension “.py” were run in Python 3. Please cite this study in any use or adaptation of this code.
