# Supplementary material for: Natural language processing methods are sensitive to sub-clinical linguistic differences in schizophrenia spectrum disorders
Source: NPJ Schizophr. 2021 May 14;7:25. doi: 10.1038/s41537-021-00154-3 (PMC8121795; doi:10.1038/s41537-021-00154-3)
Supplement: Supplementary file 1 — Supplemental Materials [file 41537_2021_154_MOESM1_ESM.pdf]

Natural Language Processing Methods are Sensitive to Sub-Clinical Linguistic Differences in Schizophrenia Spectrum Disorders

Sunny X. Tang, Reno Kriz, Sunghye Cho, Suh Jung Park, Jenna Harowitz, Raquel E. Gur, Mahendra T. Bhati, Daniel H. Wolf, João Sedoc, Mark Y. Liberman

Supplemental Materials

Supplemental Table 1: SSD Sample Details

Cohort 1		Cohort 2	
SSD (n)	15	SSD (n)	5
SAPS mean ± SD	12.1 ± 11.2	PRIME total mean ± SD	20.8 ± 18.7
SAPS range	0 - 31	PRIME total range	0 - 41
SANS mean ± SD	34.3 ± 27.1	CAINS MAP mean ± SD	1.7 ± 0.4
SANS range	0 - 98	CAINS MAP range	1.2 - 2.1

Note: All participants with SSD were stable outpatients on antipsychotic medications. Doses and medication names were not recorded. In Cohort 1, positive symptoms were rated on the Scale for the Assessment of Positive Symptoms (SAPS)[*] and negative symptoms were rated on the Scale for the Assessment of Negative Symptoms (SANS).[†] In Cohort 2, positive symptoms were reported by self-report via the PRIME screen,[‡] and avolition/anhedonia negative symptoms were evaluated with the Clinical Assessment Interview for Negative Symptoms (CAINS).[§] SD – Standard deviation.

[*] Andreasen, N.C., 1984. Scale for the assessment of positive symptoms (SAPS). University of Iowa, Iowa City.
[†] Andreasen, N.C., 1984. Scale for the Assessment of Negative Symptoms (SANS). University of Iowa, Iowa City.
[‡] Miller, T.J., McGlashan, T.H., Rosen, J.L., Somjee, L., Markovich, P.J., Stein, K., Woods, S.W., 2002. Prospective diagnosis of the initial prodrome for schizophrenia based on the structured interview for prodromal syndromes: Preliminary evidence of interrater reliability and predictive validity. Am. J. Psychiatry 159, 863–865. https://doi.org/10.1176/appi.ajp.159.5.863
[§] Kring, A.M., Gur, R.E., Blanchard, J.J., Horan, W.P., Reise, S.P., 2013. The Clinical Assessment Interview for Negative Symptoms (CAINS): Final development and validation. Am. J. Psychiatry 170, 165–172. https://doi.org/10.1016/j.schres.2011.06.030

Supplemental Table 1: Thought Language and Communication Scale Details

	HC	SSD	p value	Cohen's d
**Items: Mean (SD)**				
1. Poverty of Speech	0.00 (0.00)	0.10 (0.31)	0.29	0.40
2. Poverty of Content of Speech	0.00 (0.00)	0.25 (0.44)	0.07	0.70
3. Pressure of Speech	0.00 (0.00)	0.10 (0.45)	0.47	0.28
4. Distractible Speech	0.00 (0.00)	0.00 (0.00)	1.00	0.00
5. Tangentiality	0.27 (0.65)	0.20 (0.89)	0.81	0.09
6. Derailment	0.00 (0.00)	0.20 (0.62)	0.29	0.40
7. Incoherence	0.00 (0.00)	0.25 (0.64)	0.21	0.48
8. Illogicality	0.00 (0.00)	0.30 (0.73)	0.19	0.51
9. Clanging	0.00 (0.00)	0.05 (0.22)	0.47	0.28
10. Neologisms	0.00 (0.00)	0.10 (0.31)	0.29	0.40
11. Word Approximations	0.00 (0.00)	0.20 (0.52)	0.22	0.47
12. Circumstantiality	0.18 (0.40)	0.25 (0.72)	0.77	0.11
13. Loss of Goal	0.00 (0.00)	0.10 (0.45)	0.47	0.28
14. Perseveration	0.00 (0.00)	0.05 (0.22)	0.47	0.28
15. Echolalia	0.00 (0.00)	0.00 (0.00)	1.00	0.00
16. Blocking	0.00 (0.00)	0.10 (0.31)	0.29	0.40
17. Stilted Speech	0.00 (0.00)	0.10 (0.45)	0.47	0.28
18. Self-Reference	0.00 (0.00)	0.15 (0.49)	0.32	0.38

Note: SD – standard deviation; HC – healthy control participants; SSD – participants with schizophrenia spectrum disorders.

TLC Total Score = SUM(Items 12-18) + 2*SUM(Items 1-11)

Supplemental Table 2: Schizophrenia Spectrum and Healthy Control -Associated Words

Top SSD-Associated Words				Top HC-Associated Words			
Token	SSD Frequency	HC Frequency	Weighted Log-Odds	Token	SSD Frequency	HC Frequency	Weighted Log-Odds
[FPS] I/me…	94.7	60.7	7.2	um	16.3	25.6	-3.8
he	7.3	1.3	4.7	[FPP] we/us	9.9	17.6	-3.8
[Incomplete Word]	4.6	1.0	3.5	like	16.1	24.6	-3.4
they	8.2	4.3	2.7	of	10.6	17.0	-3.2
no	3.6	1.2	2.5	actually	0.6	2.4	-2.8
[Identifying Name]	3.8	1.5	2.3	[Laughter]	0.6	2.3	-2.7
[SP] you/your…	18.7	13.7	2.3	so	10.0	15.3	-2.7
lived	1.4	0.2	2.1	sort	0.1	1.2	-2.6
uh	17.3	12.4	2.1	usually	0.3	1.7	-2.6
well	3.9	1.7	2.1	ago	0.3	1.7	-2.5
used	2.1	0.6	2.1	great	0.3	1.3	-2.2
on	6.5	3.9	2.0	awesome	0.0	0.6	-2.2
cause	1.7	0.5	1.9	super	0.0	0.6	-2.2
him	1.4	0.4	1.9	bunch	0.0	0.6	-2.0
know	13.2	9.9	1.8	as	1.6	3.2	-2.0
people	2.7	1.2	1.8	gone	0.0	0.5	-2.0
never	1.7	0.7	1.7	wife	0.0	0.5	-2.0
had	4.5	2.6	1.6	places	0.1	0.8	-2.0
mom	1.7	0.7	1.6	recently	0.1	0.7	-2.0
florida	0.6	0.1	1.6	definitely	0.0	0.6	-1.9

Note: Tokens were weighted based on the informative Dirichlet prior (Monroe et al., 2008), which discounts words with low counts because these are likely to be statistically unreliable.

Odds ratios were calculated for words spoken by individuals with schizophrenia spectrum disorders (SSD) relative to health control participants (HC). The odds ratios were log-transformed and underwent weighting based on informative Dirichlet prior, which takes into account the expected frequency of each word in a random text and selects for words that are more "unique" to these documents. Frequency per 1000 words is reported for the 20 words/tokens most associated with SSD and with HC.

- [FPS] – first person singular pronouns, including contractions. E.g. I, I'm, I've, me, my
- [Incomplete Word] – corresponds to utterances where the word was not completely spoken. E.g. "We ***wen-***, we went to the store." "The dog bit the ***ca-***, the tabby cat."
- [SP] – second person pronouns, including contractions. E.g. you, your, you're, you've
- [FPP] – first person plural pronouns, including contractions. E.g. we, us, we've, we're, our
- [Laughter] – intervening laughter

Supplemental Figure 1: Relative Frequencies of First-Person Pronouns and Filler Words

A) First-Person Singular vs. Plural Pronouns

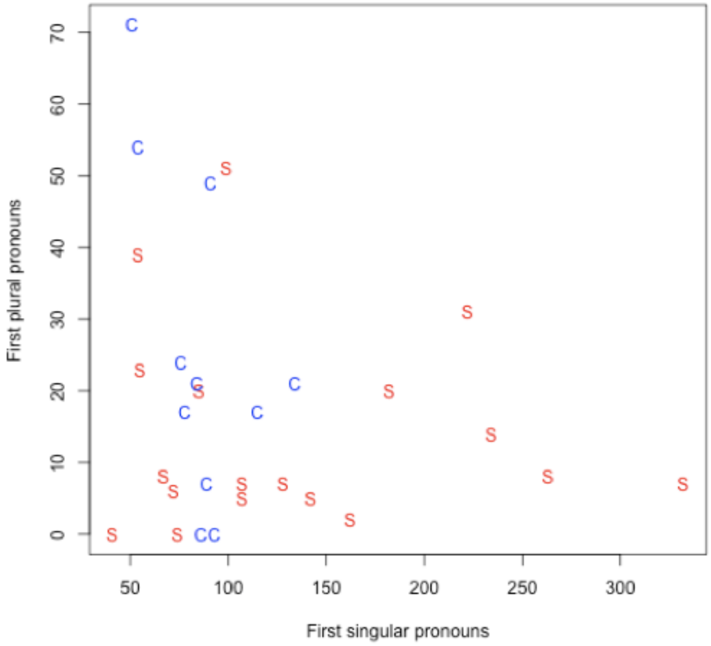

B) Filler Words: "Um" vs. "Uh"

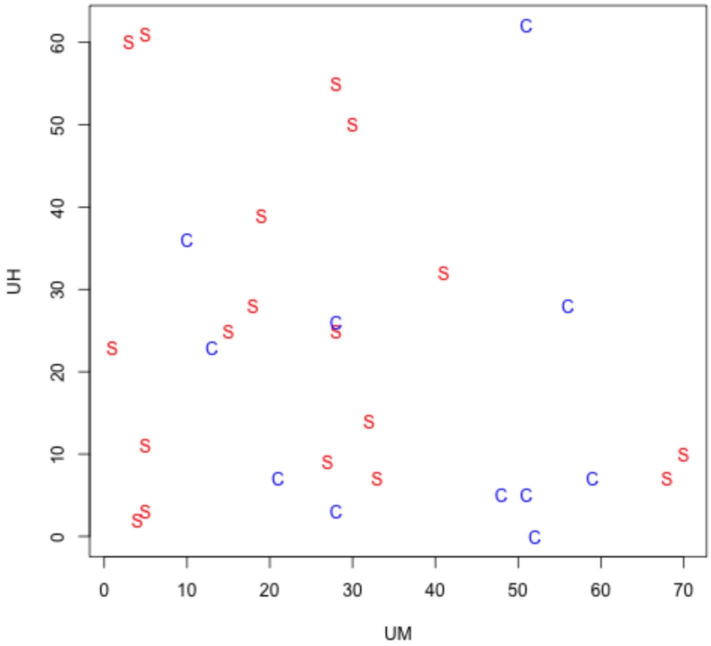

Relative frequencies of A) first-person singular and first-person plural pronouns as well as B) "Um" and "Uh" filler words are plotted for participants with schizophrenia spectrum disorders (S) and healthy control (C) participants. SSD was associated with first-person singular over plural pronouns, and the filler word "uh" over "um."

Supplemental Figure 2. Individual Words and Discrimination between SSD and HC Groups

A) Incomplete Words Only

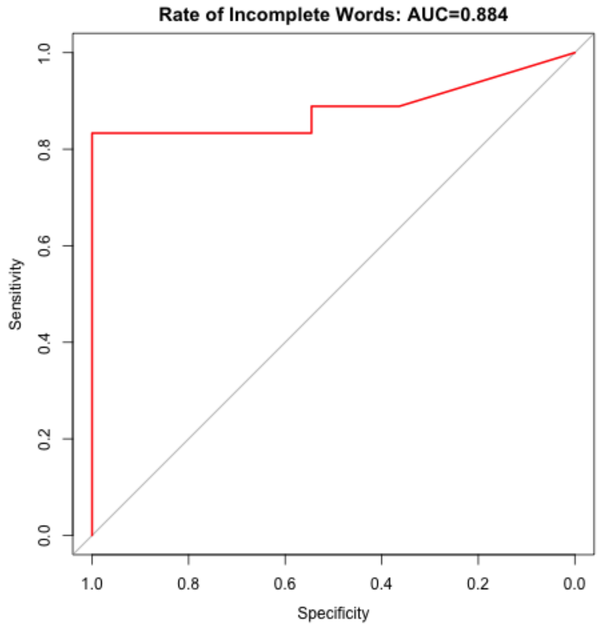

B) All Words (Leave-One-Out Cross Validation)

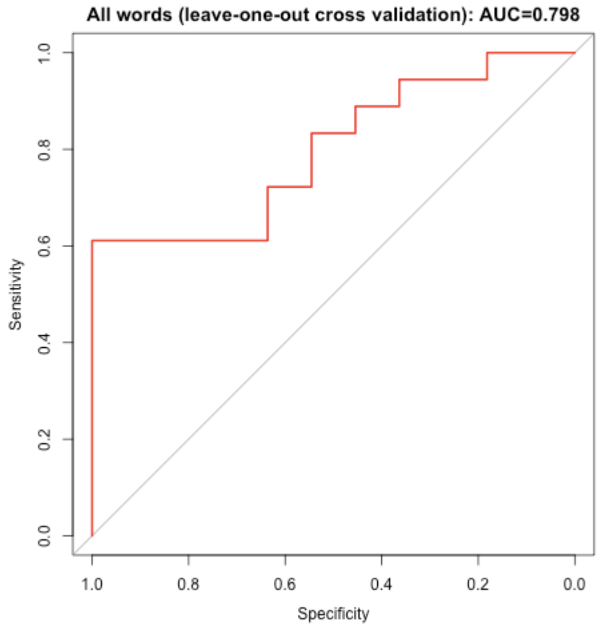

Naïve Bayes models were constructed to discriminate between SSD and HC groups using A) Only the prevalence of incomplete words, and B) the prevalence of all words.

Supplemental Figure 3: Parts of Speech in Schizophrenia-Spectrum versus Healthy Control Transcripts

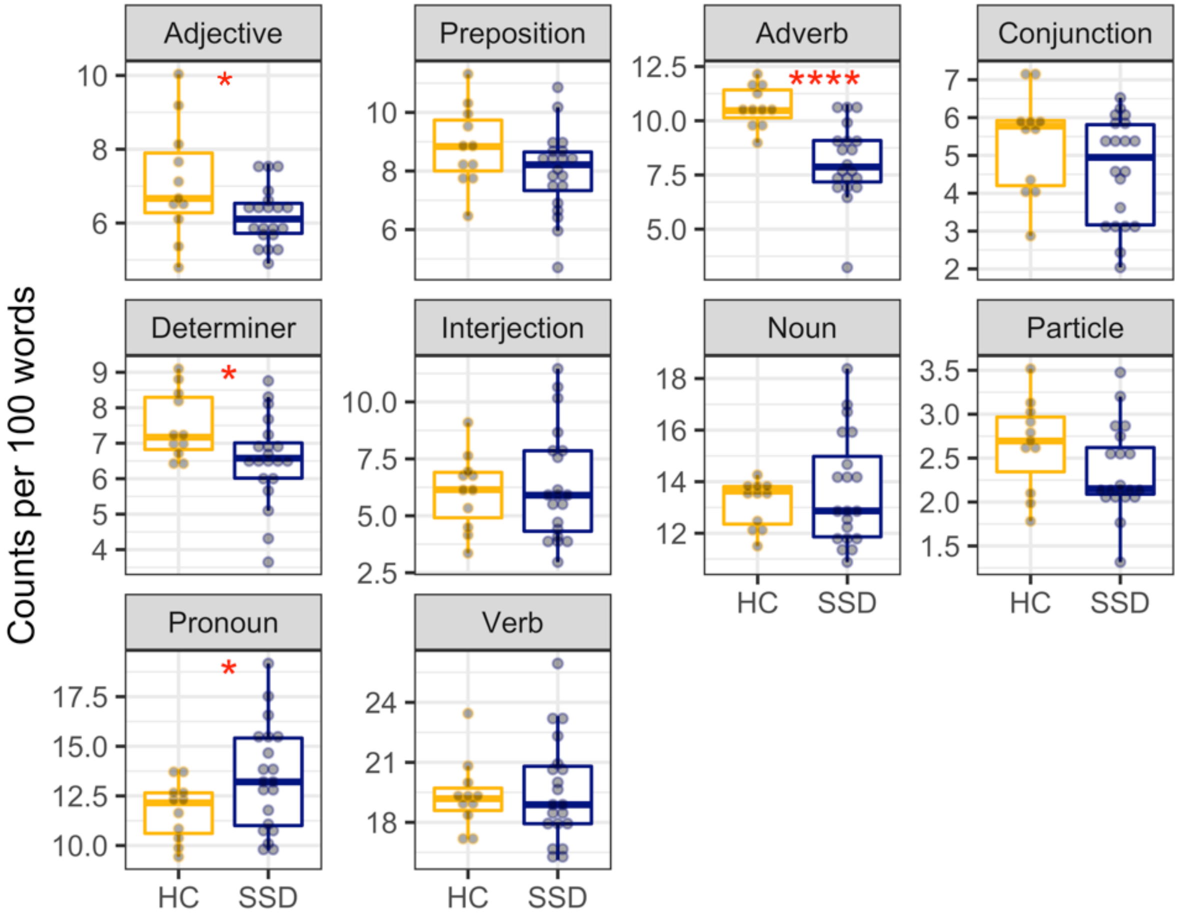

Note: HC – healthy control participants; SSD – participants with schizophrenia spectrum disorders. Adverb – p=0.001, Cohen's D=1.66; Adjective - p=0.03, Cohen's D=0.82; Determiner – p=0.03, Cohen's D=0.83; Pronoun – p=0.03, Cohen's D=-0.71.
